# Antimicrobial peptaibols, novel suppressors of tumor cells, targeted calcium-mediated apoptosis and autophagy in human hepatocellular carcinoma cells

**DOI:** 10.1186/1476-4598-9-26

**Published:** 2010-02-02

**Authors:** Mei Shi, Hong-Na Wang, Shu-Tao Xie, Yan Luo, Cai-Yun Sun, Xiu-Lan Chen, Yu-Zhong Zhang

**Affiliations:** 1 State Key Lab of Microbial Technology, Shandong University, Shanda South Road 27, Jinan 250100, PR China

## Abstract

**Background:**

Hepatocellular carcinoma (HCC) is one of the most common cancers in the world which is highly chemoresistant to currently available chemotherapeutic agents. Thus, novel therapeutic targets are needed to be sought for the successful treatment of HCC. Peptaibols, a family of peptides synthesized non-ribosomally by the *Trichoderma *species and other fungi, exhibit antibiotic activities against bacteria and fungi. Few studies recently showed that peptaibols exerted cytotoxicity toward human lung epithelial and breast carcinoma cells. However, the mechanism involved in peptaibol-induced cell death remains poorly understood.

**Results:**

Here, we showed that Trichokonin VI (TK VI), a peptaibol from *Trichoderma pseudokoningii *SMF2, induced growth inhibition of HCC cells in a dose-dependent manner. It did not obviously impair the viability of normal liver cells at lower concentration. Moreover, the suppression of cell viability resulted from the programmed cell death (PCD) with characteristics of apoptosis and autophagy. An influx of Ca^2+ ^triggered the activation of μ-calpain and proceeded to the translocation of Bax to mitochondria and subsequent promotion of apoptosis. On the other hand, typically morphological characteristics consistent with autophagy were also observed by punctate distribution of MDC staining and the induction of LC3-II, including extensive autophagic vacuolization and enclosure of cell organelles by these autophagosomes. More significantly, specific depletion of Bak expression by small RNA interfering (siRNA) could partly attenuate TK VI-induced autophagy. However, siRNA against Bax led to increased autophagy.

**Conclusion:**

Taken together, these findings showed for the first time that peptaibols were novel regulators involved in both apoptosis and autophagy, suggesting that the class of peptaibols might serve as potential suppressors of tumor cells.

## Background

Hepatocellular carcinoma is the fifth most common solid tumor worldwide and the fourth leading cause of cancer-related death. Although the majority of tumors initially respond to chemotherapy, hepatocellular carcinoma is well known for its expression of the multidrug resistance gene and its poor response to currently available chemotherapeutic agents [1,2]. Therefore, it is necessary to intensify our efforts to understand better and develop novel treatment for hepatocellular carcinoma. Promoting PCD is a strategy for cancer drug discovery. Thus there is still a significant need to explore novel antitumor agents targeted for cancer therapy and to identify mechanism-based regulators potentially useful in future clinical applications [3,4]. Peptaibols, a large family of antibiotic peptides, have been identified mainly from fungi of the genera *Trichoderma *and *Gliocladium *[5]. At present, the sequences of 309 peptaibols are known, 184 sequences of which are from the genus *Trichoderma *http://www.cryst.bbk.ac.uk/peptaibol/home.shtml[6,7]. The most common researches on peptaibols emphasize the biosynthetic pathway, conformational properties, determination of amino acid sequences and biological activities. However, there are few reports about the effects of peptaibols on human cells or cancer cells [8,9]. Peltola [10] reported that the peptaibols from *Trichoderma harzianum *suppressed the growth of A549 cells and dissipated the mitochondrial membrane potential (ΔΨ_m_). However, the precise mechanism and the basic components in cellular death pathway involved in peptaibol are currently unknown.

Under physiological and pathological settings, PCD can be classified into several morphological and biochemical subtypes. Among them, the most prominent types are type-1 cell death (apoptosis) and type-2 cell death (autophagic cell death, ACD). Recent reviews have described the relationship of autophagy and apoptosis signaling during cancer therapy. While the molecular mechanisms leading to apoptosis have been dissected to some extent during the past 15 years, ACD is not well characterized at the molecular level yet [11]. Autophagy has recently gained much attention for its especial roles in cell survival and cell death, particularly in the pathogenesis as well as the treatment of cancer. Not surprisingly, there is an intricate relationship between autophagy and apoptosis. Early studies showed that autophagy took over when apoptosis was blocked in cancer cells. A more recent report demonstrated that autophagy and apoptosis might interact or occur independently of each other [12,13]. Therefore, the interplay between autophagy and apoptosis is unclear. A debate issue is whether crosstalk between these two pathways exists and how cell death declines to one or the other subroutine.

Previous studies have indicated that the elevation of [Ca^2+^]_*i *_was a sufficient signal to induce apoptosis in several model systems [14]. It is important to elucidate whether cells undergo apoptosis, autophagy, or both, in the response to calcium elevation [15]. Calpain is an intracellular cysteine protease that modulates Ca^2+^-dependent apoptosis through a variety of mechanisms. The best-characterized calpains are two ubiquitously expressed isozymes, μ-calpain and m-calpain, sharing homology in their protease domains. To become active, calpains require an elevation in [Ca^2+^]_*i*_, and the autoproteolytic cleavage to further enhance their activity. Recent studies have indicated that calpains may play a central role in the execution of calcium-triggered cell death upstream of caspases. Moreover, calpain has been implicated in the translocation of Bax to the mitochondria. In addition, calpain has been reported to cleave Bid, a BH3-only Bcl-2 family protein, resulting in the cytochrome *c *(Cyto-*c*) release from mitochondria [16-18]. It has also been suggested that Bcl-2 family members (Bax/Bak and Bcl-2/Bcl-xL) could promote or inhibit the apoptosis and autophagy presumably through an alternate mechanism [15,19]. It is not well known that a cell decide whether to undergo apoptosis, autophagy, or both, in the response to calcium elevation and the role of Bcl-2 family members in regulating calcium signals and proceeding of autophagy.

Trichokonins (TKs) secreted by *T. koningii *are peptaibols which are composed of 20 amino acids. Three TKs, TK VI, TK VII and TK VIII, have been isolated from *Trichoderma *spp. SMF2, and their sequences have been identified [20-22]. Strain SMF2 was described as *T. koningii *in our previous reports [22]. However, through analyses of its colony, conidiophore morphology, 18S rDNA and ITS sequences (Gene Bank accession number FJ605099), strain SMF2 was recently identified as *T. pseudokoningii *(see Additional file 1). Like other peptaibols, TKs have been shown to have antibacterial and antifungal properties. In this paper, we identified TK VI as a novel cell death regulator by studying its cytotoxic effects on cancer cells and HCC cells (HepG2 cells). Our results first provided molecular evidence to demonstrate a novel finding that TK VI induced two types of cell death, including calcium-calpain-Bax-mediated apoptosis and calcium-Bak-mediated autophagy in HepG2 cells.

## Results

### TK VI predisposed cancer cells to apoptosis accompanied by morphological changes compatible with autophagy

**Figure 1:**
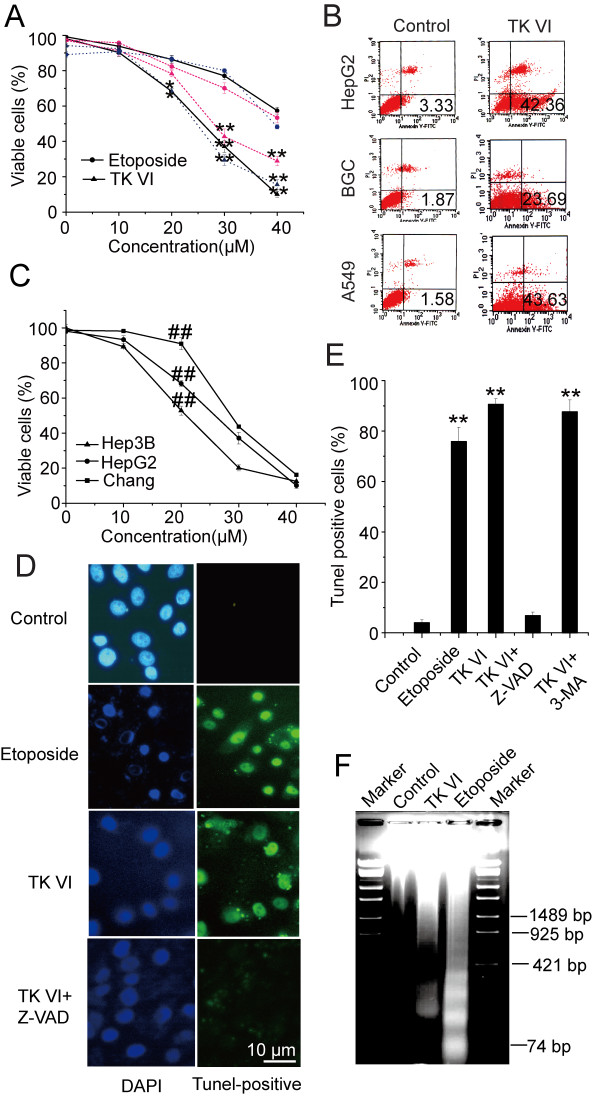
**Assessment of cell death and apoptosis induced by TK VI**. (A) Cells were treated with TK VI or etoposide, ranging from 10 to 40 μM and incubated in 10% FBS-DMEM complete medium at 37°C for 24 h. Viable cells were measured by MTT assay. Results were expressed as means ± SD of triplicate experiments (each performed in duplicate). Black solid line: HepG2 cells; Red short dash line: BGC cells; Blue dot line: A549 cells. Asterisks indicated values significantly different from cells treated with etoposide. *P < 0.05; **P < 0.01. (B) Cells were treated with 20 μM TK VI for 8 h and then apoptosis were analyzed on a flow cytometer using annexin V/PI staining methods. Data were representative of three independent experiments. Numbers in the respective quadrants indicated the percentage of the cells present in this area. (C) The HepG2, Hep3B and Chang liver cells were incubated with TK VI and detected as described in A. Results were expressed as means ± SD of triplicate experiments (each performed in duplicate). ## indicated values significantly different from Chang liver cells (^##^P < 0.05). (D) HepG2 cells were treated with 20 μM TK VI or etoposide for 24 h. Then DNA fragmentation in the cells was viewed using fluorescence microscopy. (E) The quantitative analysis of apoptosis in cells was analyzed by FACS using TUNEL staining. Results were expressed as means ± SD of triplicate experiments (each performed in duplicate). Asterisks indicated values significantly different from controls and Z-VAD-treated cells. **P < 0.01. (F) DNA was extracted from HepG2 cells cultured for 36 h with 20 μM TK VI and DNA ladder was detected by agarose gel electrophoresis.

To assess whether TK VI induced cell death, MTT assay was used to measure the relative survival of cells. When BGC-823, A549 and HepG2 cells were incubated with increasing concentrations of TK VI, TK VI and etoposide caused a dose-dependent inhibition of cell proliferation, whereas the inhibitory effect of TK VI was approximately two-fold higher than that of etoposide with concentrations ranging from 30 to 40 μM (Figure. 1A). Further cytometric analysis using FITC-labeled annexin V showed that all three cell lines treated with 20 μM TK VI for 8 h exhibited significant increases in apoptosis (Figure 1B).

Whether TK VI had different effects on hepatocellular carcinoma and normal liver cells were further determined. The human hepatocellular carcinoma cell lines HepG2, Hep3B and human liver cell line Chang were treated with increasing concentration of TK VI. As shown in figure 1C, TK VI caused a dose-dependent inhibition on cell proliferation of three cell lines with concentrations ranging from 10 to 40 μM. However, at lower concentration, TK VI did not obviously impair the viability of normal liver cells. Moreover, a 3' end labeling of DNA assay in HepG2 cells using fluorescence microscopy showed positive results (Figure 1D). The percentage of HepG2 cells showing apoptosis (positive TUNEL reaction) was further determined by FACS (Figure 1E). Of the cells treated with 20 μM TK VI and etoposide for 24 h, about 90% and 80% of HepG2 cells showed apoptosis, respectively. However, the apoptotic populations in cells treated with TK VI and Z-VAD-fmk were dramatically decreased in contrast to cells treated TK VI alone. In addition, 3-MA had no effect on TK VI-induced apoptosis. Nevertheless, compared with etoposide-treated cells, DNA laddering was less pronounced in TK VI- treated cells (Figure 1F). These results indicated that TK VI-treated cells displayed a significant apoptotic character in a caspase-dependent manner.

**Figure 2:**
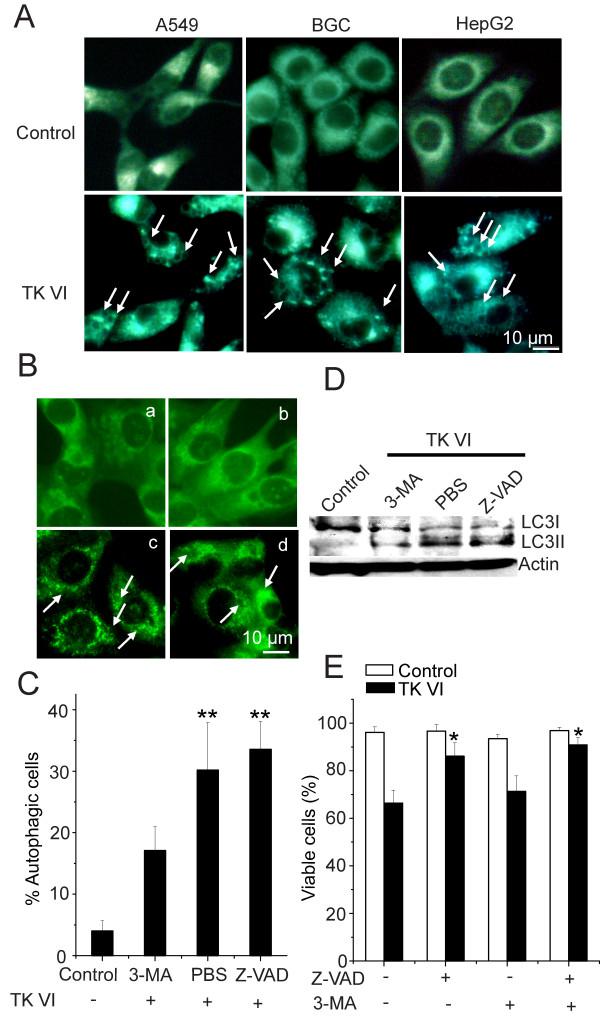
**Assessment of cell death and autophagy induced by TK VI**. (A) Monodansycadaverine (MDC)-labeled vesicles (indicated by arrows) were induced by TK VI. HepG2, BGC and A549 cells were incubated in DMEM medium (control cells) or treated with 20 μM TK VI and etoposide at 37°C for 24 h. Cells were immediately analyzed by fluorescence. (B) Iimmunocytochemistry for LC3 localization in HepG2 cells treated for 16 h with TK VI alone and combined with Z-VAD-fmk or 3 MA. a: control; b to d: cell treated with TK VI+3-MA, TKVI, and TK VI +Z-VAD-fmk. (C) Cells were treated the same as decribed in (B) and analyzed by acridine orange staining using flow cytometry for autophagy. Results were expressed as means ± SD of triplicate experiments (each performed in duplicate). Asterisks indicated values significantly different from controls and 3-MA-treated cells. **P < 0.01. (D) Cells were treated the same as decribed in (B). Cell lysates were analyzed using Western blotting with anti-LC3 and -actin antibodies. β-actin was used as an internal control to normalize the amount of proteins applied in each lane. (E) HepG2 cells were pretreated with 3-MA or Z-VAD-fmk for 1 h, followed by treatment with 20 μM TK VI for 24 h to determine cell viability using MTT assay. Asterisks indicate values significantly different from cells only treated with TK VI. *P < 0.05.

It has been reported MDC as a specific marker for autolysosomes [23]. Therefore, we studied the incorporation of MDC in the cells where autophagy was stimulated by TK VI using fluorescence microscopy. As shown in Figure 2A, cells treated with TK VI for 24 h showed an increase in the number of vesicles as well as in their size, indicating the formation of the MDC-labeled vacuoles. MDC was concentrated in the spherical structures distributed in the cytoplasm and also localized in the perinuclear region. LC3 exists as two forms, such as an 18 kDa cytosolic protein (LC3-I) and a processed 16 kDa form (LC3-II) in the cells engaged in autophagy. Studies have also revealed that the LC3-II form is mainly localized in autophagosome membranes [24]. In order to test the involvement of LC3 in TK VI-induced autophagy, we initially determined the effect of TK VI treatment on the localization of LC3 by immunocytochemical analysis and the results are shown in Figure 2B. The control HepG2 cells exhibited diffused distribution of LC3-associated green fluorescence. On the other hand, HepG2 cells treated with TK VI for 16 h displayed a punctate pattern of LC3-II immunostained with increased fluorescence, which characterized its redistribution to autophagosomes. By contrast, in the presence of 3-MA, an autophagy-specific inhibitor, autophagy was prevented effectively. Moreover, the pretreatment of cells with Z-VAD-fmk produced a slightly enhanced punctate pattern of LC3-II fluorescence, indicating the blockage of apoptosis could mildly promote TK VI-mediated autophagy (Figure 2B). The percentage of autophagic cells was further determined by FACS using staining with an acridine orange fluorescence dye and the results were similar to Figure 2B (Figure 2C). Next, we tested whether TK VI treatment caused proceeding of full-length LC3-I (18 kDa) to LC3-II (16 kDa) by performing immunoblotting using lysates from HepG2 cells treated with TK VI alone or combined with z-VAD-fmk and 3-MA (Figure 2D). The results were consistent with Figure 2B. These results collectively suggested that treatment with TK VI specifically and efficiently induced autophagy in HepG2 cells. More importantly, using MTT assay to monitor the effects of 3-MA on cell viability (Figure 2E), we observed that after 24 h of 20 μM TK VI exposure, the percentage of viable cells increased, but there was relatively minor effect. On the other hand, the proportions of viable HepG2 cells after treatment with TK VI significantly increased from 66% to 86% in the presence of Z-VAD-fmk. Furthermore, co-treatment with Z-VAD-fmk and 3-MA had additive effects on cell viability. Taken together, TK VI induced two types of cell death in HepG2 cells, including both apoptosis and autophagy and the TK VI-induced apoptosis mainly attributed to cell death.

### TK VI increased [Ca^2+^]_*c *_in a time and dose-dependent manner

**Figure 3:**
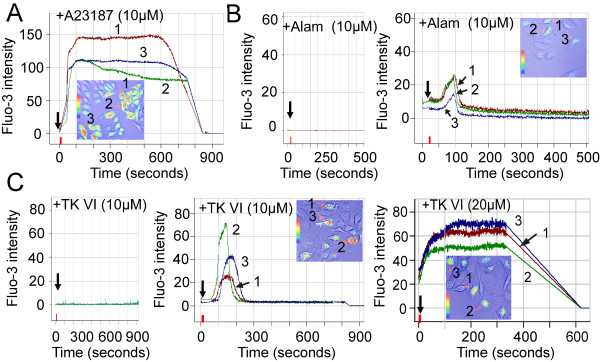
**Assessment of TK VI-induced [Ca^2+^]_*c *_increases using confocal fluo-3 fluorescence imaging**. (A)A23187 was a positive control for agitation of [Ca^2+^]_*c*_. (B) and (C), HepG2 cells were loaded with Fluo-3 acetoxymethyl ester and treated with 10 and 20 μM TK VI or Alamethicin (ALam, a classical representative of peptaibols) diluted in PBS alone or containing 2 mM CaCl_2_. Then cells were examined from 0 to 900 sec for Fluo-3 fluorescence intensity. Original trace of fluorescence intensity as a measure of [Ca^2+^]_*c *_recorded from the cells was marked in the confocal images. TK VI-induced [Ca^2+^]_*c*_ increase was assessed by using confocal two-photon fluo-3 fluorescence imaging. (B: right panel and C: middle and right panel). Three cells responded to TK VI stimulation with an increase in fluo-3 fluorescence intensity were used as a measure of [Ca^2+^]_*c *_(indicated with arrows). The graph was labeled with arabic numbers that referred to the images. The images of cells treated with TK VI and Alamethicin (10 μM) diluted in PBS alone were shown in left panels in B and C.

The elevation in [Ca^2+^]_*c *_due to the influx of Ca^2+ ^across the cell membrane or the release of intracellular Ca^2+ ^stores, may mediate apoptosis [25]. Cells were treated with A23187, a classical calcium carrier, as a positive control (Figure 3A). To investigate whether TK VI and Alamethicin led to the [Ca^2+^]_*c *_increase, [Ca^2+^]_*c *_measurements were performed using laser scanning microscopy. [Ca^2+^]_*c *_was measured confocally on the single-cell level with fluo-3. Typical fluo-3 fluorescence images (recorded at 780-nm excitation) were presented at indicated times (Figure 3B and 3C). When HepG2 cells were exposed to 10 μM Alamethicin or TK VI in medium containing 2 mM CaCl_2_, about one-third the cells in the image displayed high fluorescence intensity (Figure 3B, right panel and Figure 3C, middle panel). While HepG2 cells were exposed to 10 μM TK VI or Alamethicin in the medium without the addition of Ca^2+^, the Fluo-3 intensity did not appreciably alter in the cells (Figure 3B, left panel and Figure 3C, left panel). The fluo-3 fluorescence traces from the representative cells are presented. The results indicated that TK VI promoted an increase in [Ca^2+^]_*c *_in a time-dependent manner, which originated from an influx of extracellular Ca^2+^. When the concentration of TK VI was increased from 10 to 20 μM, the intensity of fluo-3 fluorescence significantly increased with the increase of TK VI concentration (Figure 3C, right panel), indicating that TK VI treatment also increased [Ca^2+^]_*c *_in a dose-dependent manner.

### Calpain was a key factor in calcium-originated pathway in TK VI-treated cells

**Figure 4:**
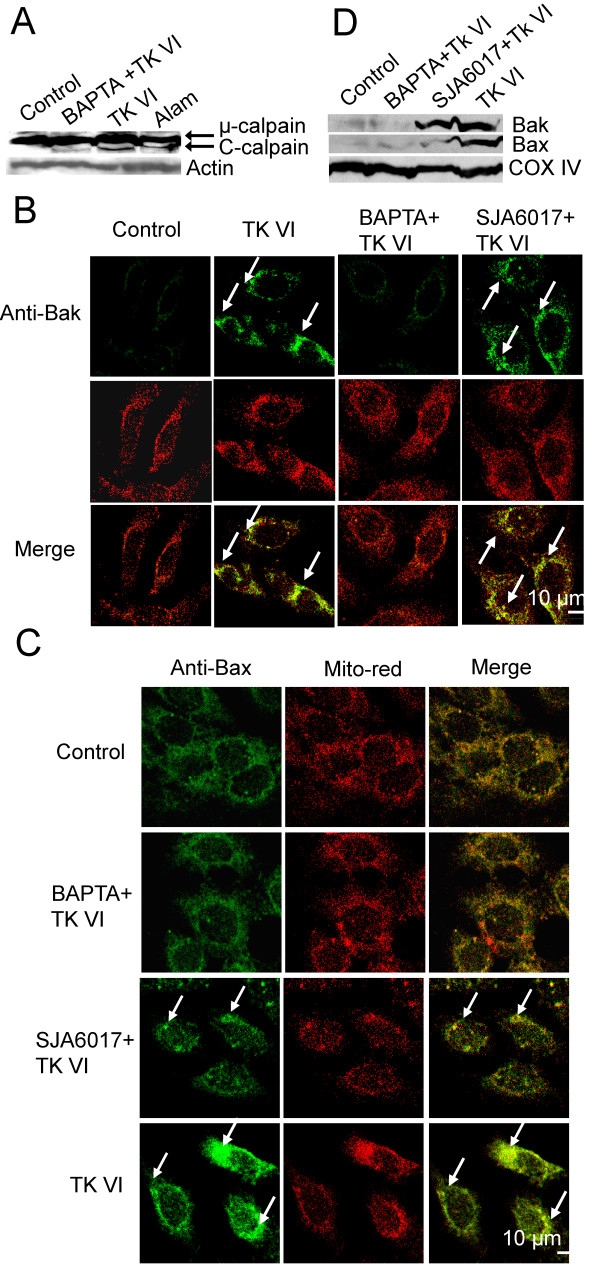
**The status of calpain in TK VI-treated cells**. (A) Protein samples derived from HepG2 cells treated with 20 μM TK VI for 8 h were analyzed by immunoblotting. The 80-kDa pro-form of μ-calpain appeared as an abundant band, and the cleaved 78-kDa form of the enzyme was visible in some samples just below the pro-form. (B) HepG2 cells were pretreated with BAPTA and SJA6017, followed by 20 μM TK VI for 8 h. Then activation of Bak was detected with activated N-terminal anti-Bak antibody and mitochondria were visualized with red fluorescent protein (Mito-red) by imunofluorescence microscopy. (C) The cells were incubated with TK VI as described in (B). Mitochondria were visualized with red fluorescent protein (Mito-red). The redistribution of Bax was marked with FITC-conjugated anti-mouse and assessed by confocal immunofluorescence. (D) The cells were incubated with TK VI as described in (B). Equal amounts of mitochondrial proteins was isolated from HepG2 cells and then subjected to immunoblot analysis. Cox IV was used as a marker for mitochondrial fraction.

The activation of calpain is demonstrated by the autoproteolytic cleavage, which is a specific marker for calpain activation [26]. To investigate whether calcium-dependent proteases, calpains, were activated during the treatment of HepG2 cells with TK VI, the cleavage of calpain into its active form was first analyzed by immunoblot analysis. As shown in Figure 4A, the cells treated with TK VI and Alamethicin contained a cleaved form of μ-calpain. Calpains can be upstream mediators of apoptosis by triggering the mitochondrial death program through the activation of some Bcl-2 family members. Bax is critical for the breakdown of the mitochondrial potential by translocating to the mitochondria after a cell receives death stimuli [27]. Therefore, Bak and Bax activation in HepG2 cells treated with TK VI was examined by immunohistochemical method. To confirm whether Bak was activated in response to TK VI, HepG2 cells were incubated with a unique antibody that was only accessible to activated Bak. It was observed that treated cells displayed a bright, punctuate staining pattern of Bak antibody after 8 h treatment (Figure 4B), while this pattern was not seen in control cells. In the cells pre-treated with BAPTA, Bak remained inactive after TK VI treatment. On the contrary, inhibition of calpain by SJA6017, calpain inhibitors, had no effects on the activation of Bak, indicating that activation of Bak was only enhanced in the presence of [Ca^2+^]_*c*_, but not calpain. Furthermore, consistent with the previous reports [26,27], our results showed that Bax translocated to the mitochondria and redistributed in the mitochondria after TK VI treatment. However, if the cells were pre-treated with BAPTA or SJA6017, Bax was still located in the cytosol of the cells and remained inactive after TK VI treatment (Figure 4C). Under the same conditions, the results of immunoblot analysis were similar to that of the immunohistochemical detection (Figure 4D). These results indicated that activated calpain induced Bax activation and translocation to mitochondria after TK VI treatment.

### Bak played as a major factor in calcium-originated autophagic cell death induced by TK VI

**Figure 5:**
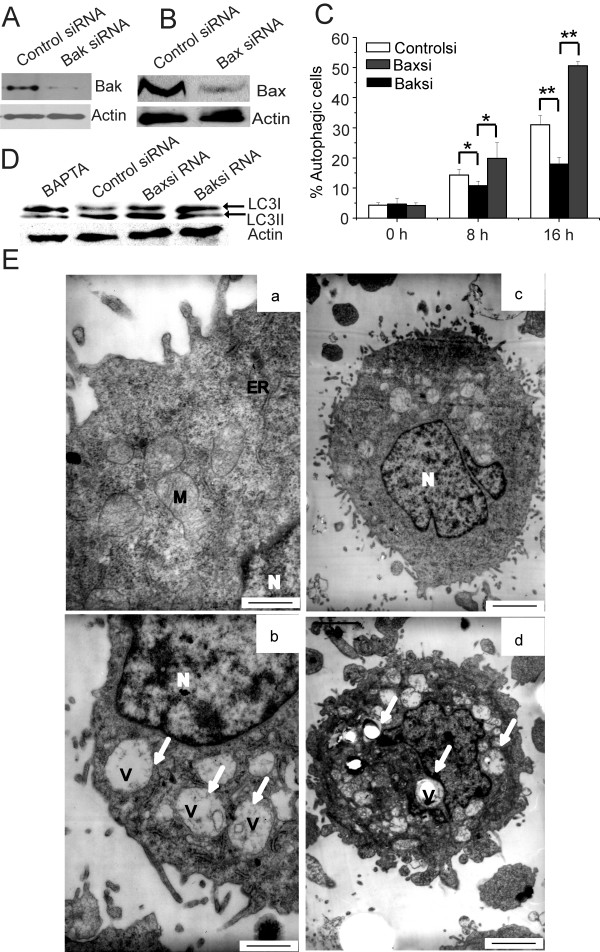
**siRNAs against Bax enhanced autophagy in HepG2 cells treated with TK VI**. (A) and (B) HepG2 cells were transfected with Bak siRNA and Bax siRNA or control siRNA. After 48 h, the expression of Bak and Bax in interfered and control cells were detected by Western analysis. (C) Cells were treated with 20 μM TK VI for indicated time and then autophagy was analyzed using flow cytometry with acridine orange dyeing. Asterisks indicated values significantly different from Bak siRNA cells. *P < 0.05; **P < 0.01. (D) Cells were incubated with 20 μM TK VI for the 16 h. Cell lysates were analyzed using Western blotting with anti-LC3. (E) Morphological changes of autophagic cell death at 24 h of TK VI exposure. a: Control cells; b: Control siRNA cells treated with 20 μM TK VI. c and d: Bak siRNA and Bax siRNA cells treated with 20 μM TK VI, respectively. *N*, Nucleus; *ER*, endoplasmic reticulum; *M*, mitochondria; *V*, vacuole. Extensive cytoplasmic vacuolization was seen in TK VI-treated cells (indicated by arrows). The images with high magnification showed that the autophagic vacuoles contained electron dense material and degraded subcellular organelles. Bars represented 1.4 μm (a), 0.8 μm (b), 0.5 μm (c, d).

Previous studies showed that Bak was a typical cell death determinant, and Bcl-2 famlily proteins were essential links between apoptosis and autophagy [28]. A Bak and Bax-interfered cell line (Bak siRNA cells and Bax siRNA cells) was constructed from HepG2/Bak^+/+ ^and HepG2/Bax^+/+ ^cells, and reduction of Bak and Bax expression in cells were detected by immunoblot analysis, respectively (Figure 5A and 5B). In this study, we observed that control cells underwent visible autophagy after treatment with TK VI for 16 h, but Autophagy was obviously enhanced in Bax siRNA cells compared with control cells. Unexpectedly, Bak siRNA cells exerted a remarkable decrease in autophagy (Figure 5C). Next, we tested whether Bak siRNA and Bax siRNA cells treated with TK VI caused proceeding of full-length LC3-I to LC3-II, a hallmark of autophagy. As shown in Figure 5D, the levels of LC3-II proteins dramatically increased after treatment with TK VI in Bax siRNA cells. In the meantime, cells pretreated with BAPTA and Bak siRNA cells did not show corresponding increase in LC3-II proteins. Transmission electron microscopy assay confirmed control cells displayed normal cell morphology (Figure 5E, a). Approximately 30-35% of the TK VI-treated cells developed vacuoles after 24 h treatment, some of which accumulated to form larger vacuoles (Figure 5E, b). At higher magnifications, most vacuoles contained electron dense material and degraded organelles. Morphologically, the vacuoles primarily originated from mitochondria. Most cells were strongly vacuolated, and the size of the vacuoles increased to 2-3 μm. Blockage of Bak attenuated part of TK VI-induced vacuolization effect (Figure 5E, c). In contrast, Bax-interfered cells displayed increasedly vacuolated morphology (Figure 5E, d). Taken together, the results indicated that [Ca^2+^]_*c *_was an indispensable factor in the autophagic cell death and Bak played a major role in autophagy induced by TK VI. Moreover, the inhibition of Bax could obviously enhance autophagic sensitization in cells.

### Translocation of Bax was essential for TK VI-mediatedapoptosis

**Figure 6:**
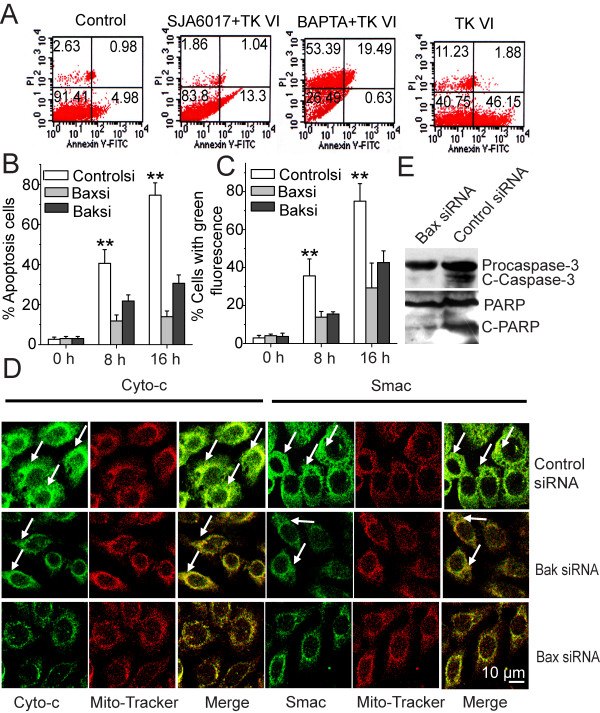
**The requirement of translocation of Bax in TK VI-induced apoptotic cell death**. (A) HepG2 cells were treated with 20 μM TK VI for 8 h, or pretreated with calpain inhibitor (SJA6017, 100 μM) and chelators of calcium (BAPTA acetoxymethyl ester, 10 mM). Subsequently, cells were assessed for the exposure of PS with annexin V staining and for viability with PI exclusion by flow cytometry (one representative of three similar experiments). Numbers in the respective quadrants indicated the percentage of the cells present in this area. (B) and (C) Apoptotic cells and dissipation of ΔΨ_m _were quantified by FACS analysis. HepG2 cells were transfected with Bax siRNA or control siRNA. HepG2 cells were treated by 20 μM TK VI for indicated time periods and stained by annexin V and Rhodamine 123. Quantification of the percentage of apoptotic cells and changes in the mitochondrial membrane potential were detected using Flow Cytometry. Asterisks indicated values significantly different from Bax siRNA cells. **P < 0.01. (D) Change of Cyto-*c *and Smac distribution was detected after 8-h TK VI (20 μM) treatment. Mitochondria were visualized with red fluorescent protein (Mito-red) and Cyto-*c *or Smac was marked with FITC-conjugated anti-mouse and anti-rabbit IgG, respectively. (E) The cleavage of procaspase-3 and PARP were detected after 16 h of TK VI treatment. Data represented means and SD. from three independent experiments.

The effect of calcium and calpain on apoptosis was first analyzed in HepG2 cells. When cells were exposed in the presence of BAPTA-AM, an effective inhibition in TK VI mediated activation of calpain was shown and TK VI-stimulated PCD was reduced (Figure 4A and 6A). In addition, the calpain inhibitor SJA6017 partially attenuated the TK VI-stimulated apoptosis. These results showed that Ca^2+ ^influx was a prerequisite in TK VI-mediated apoptosis, and subsequent activation of calpain was necessary for the process. In normal cells, the Bax protein exists as an inactive form in the cytosol, but it can be induced to change conformation and translocate to the mitochondria in the response to certain apoptotic stimuli [29]. After 24 h treatment with TK VI, most control siRNA cells underwent apoptosis, whereas little apoptosis was observed in Bax siRNA cells (Figure 6B). Even after 48-h treatment, only a few Bax siRNA cells underwent cell death (data not shown). Along with results shown in Figure 4C, the data suggested that Bax was required for TK VI-induced apoptosis in HepG2 cells.

In the course of mitochondrial apoptosis, the loss of ΔΨ_m _and the release of cytochrome *c *from the mitochondrial intermembrane space into the cytosol are the major events [30]. Flow cytometry analysis showed that TK VI-treated cells displayed a significantly higher percentage of cells with damaged mitochondria after 16 h treatment. These results showed TK VI promoted mitochondrial transmembrane potential dissipation. Interestingly, blockage of Bax drastically reduced dissipation of ΔΨ_m_(Figure 6C), and subsequently almost completely inhibited the release of cytochrome *c *and Smac (Figure 6D) in TK VI-treated cells. Moreover, after 4-h TK VI treatment, the cleaved forms of caspase-3 and PARP appeared in control siRNA cells. In contrast, cleavage of procaspase-3 and PARP was absent in Bax siRNA cells (Figure 6E). These results showed that the mitochondrial pathway, as well as activation of procaspase-3 and PARP, was induced by TK VI treatment in a Bax-dependent manner.

### Bcl-xL down-regulation was required for TK VI-inducedapoptosis

**Figure 7:**
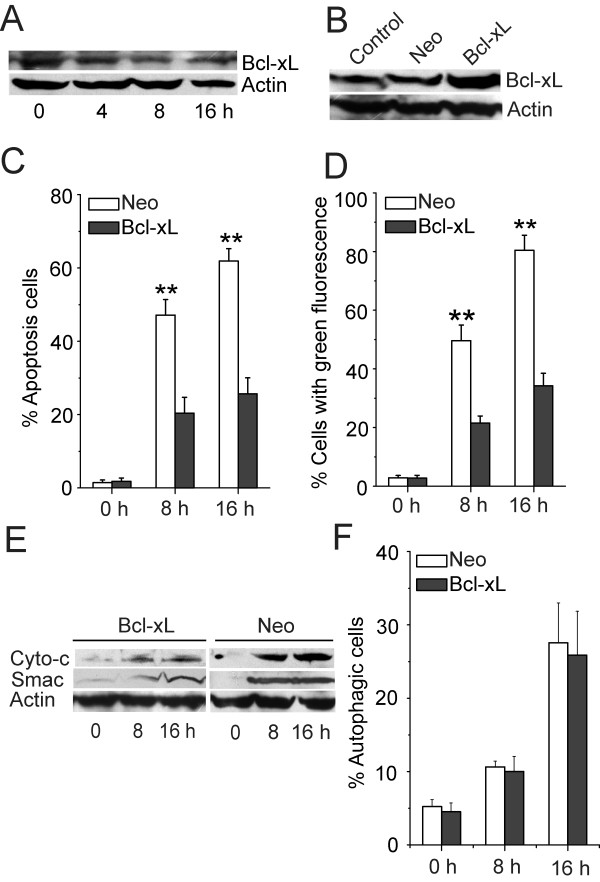
**The retardance of apoptosis with overexpression of Bcl-xL**. (A) HepG2 cells were treated by 20 μM TK VI and the expression of Bcl-xL was assessed by Immunoblot assay. (B) HepG2 cells were transfected with a pcDNA3.1 vector containing the coding sequence for Bcl-xL (Bcl-xL) or with a neomycin-resistant expression vector pcDNA3.1 (Neo, control) by Lipofectin reagent according to the manufacturer's instructions. Over-expression of Bcl-xL expression was assessed by Western blot. (C) and (D) Apoptotic cells and Dissipation of ΔΨ_m _were quantified by FACS analysis. Results were expressed as means ± SD of triplicate experiments (each performed in duplicate). (E) HepG2 cells were treated by 20 μM TK VI for indicated times. The release of Cyto-*c *and Smac/DIABLO was assessed in Bcl-xL-overexpression and neo cells by Immunoblot assay. (F) Cells were treated as (C) and then autophagy was analyzed using flow cytometry with acridine orange dyeing. Asterisks indicated values significantly different from Bcl-xL-overexpression cells. **P < 0.01.

Bcl-xL inhibits cell death by blocking the formation of the cytochrome *c*-releasing pores. Previous studies showed that Bcl-xL is down-regulated during apoptosis induced by chemotherapy reagents [31]. After treatment with TK VI, the level of Bcl-xL decreased in HepG2 cells (Figure 7A). Compared with the control/neo cells, approximately 2-fold higher level of Bcl-xL was expressed in the Bcl-xL-overexpression cells (Figure 7B). Apoptosis was induced in 60% of Control/neo cells after 16 h of TK VI treatment, but a little apoptotic cell death was observed in the Bcl-xl-overexpression cells treated with TK VI for 24 h (Figure 7C). In addition, dissipation of ΔΨm was retarded (Figure 7D), following a significant delay in the release of cytochrome *c *and Smac/DIABLO by the reason of overexpression of Bcl-xL (Figure 7E). As revealed in Figure 7F, the change in percentage of autophagic cells was almost not detected. These results confirmed that TK VI-induced apoptosis was sustained by mitochondrial release of apoptotic molecules, which triggered primarily by the regulation of Bax and Bcl-xL in HepG2 cells (Figure 6 and Figure 7).

## Discussion

Peptaibols are a family of antibiotic peptides isolated from soil fungi that exhibit anti-bacterial and anti-fungal properties [32]. Few studies show they have the capacity to induce cell death in human cancer cells [10,33,34]. In this report, we first used HepG2, BGC-823 and A549 cell lines as models for MTT assay and found that TK VI inhibited the growth of the three cell lines *in vitro*. Most significantly, HepG2 cells were more sensitive to TK VI than normal liver cells with lower concentration of TK VI (from 10 to 20 μM) (Figure 1), indicating that peptaibol might be a potentially novel anticancer agent. Furthermore, TK VI induced a form of apoptotic and autophagic cell death in HepG2 cells, which was characterized by extensive vacuolization, surface exposure of PS, and loss of mitochondrial membrane. Moreover, the apoptotic process was caspase-dependent. In addition, cell death induced by TK VI was mainly characteristic of apoptosis in HepG2 cells, whereas small interfering RNAs against Bax led to increased autophagy. Interestingly, the kinetics of Ca^2+ ^influx correlated well with the onset of apoptosis and autophagy, suggesting that Ca^2+ ^might play a direct role in these processes. Calcium caused a significant activation of Bak and thus promoted authphagic pathway in TK VI-treated cells.

Changes in intracellular Ca^2+ ^have been reported to occur at the beginning of cell death, induced by pore-forming toxins or peptides from bacteria, animals and plants. The fact that peptaibols formed ion channels in phospholipid bilayers [7] prompted us to investigate the effect of TK VI treatment on intracellular Ca^2+ ^levels. Our results suggested that TK VI initially induced an increase in [Ca^2+^]_*c *_in HepG2 cells, which originated from an influx of extracellular Ca^2+ ^(Figure 3). Peptaibols are fungal peptides rich in non-coded α-aminoisobutyric acids (Aib). The structure, sequence and amphipathic property of peptaibols play major roles in its cell-killing mechanism [35,36]. The long sequence peptaibols with 18-20 amino acid residues, exemplified by alamethicins [37], were used to explore the channel formation of these peptides on cells in the "barrel-stave" model [7,8,37]. Fonteriz reported [38] that alamethicin seemed to form ionic channels in chromaffin cells, which were permeable to Ca^2+^. Interestingly, in the view of Qing Hua [39], trichokonin VI was a potent agonist of L-type Ca^2+ ^channel. Our results demonstrated that elevation of [Ca^2+^]_*c *_attributed to TK VI and it played a crucial role not only in the apoptosis but also in the autophagy induced by TK VI. More importantly, we report for the first time that TK VI exhibited its cytotoxicity via autophagy through Ca^2+^-Bak signaling. We observed that BAPTA abolished calcium influx and significantly reduced TK VI-induced autophagy and apoptosis (Figure 5D and Figure 6A). Consistent with our results, vitamin D, ATP, ionomycin, and photodynamic therapy were known to induce calcium-mediated autophagy [40]. The involvement of the calpain, a key calcium-dependent protein, in apoptosis was suggested by previous studies involving different cellular systems [26]. Remarkably, the inhibitors of calpain abrogated the activation of Bax and further protected HepG2 cells against apoptosis induced by TK VI. It is noteworthy that calpain inhibitors conferred only a partial protection against apoptosis (Figure 4C and Figure 6A). Consistent with this observation, other effective molecules, in addition to calpains, might be involved in this apoptotic pathway. Although Bak was not an effector of calpain, but the influx of Ca^2+ ^contributed to its activation (Figure 4B). The results suggested calpain was one of the major factors in TK VI-mediated apoptosis.

The relationship of autophagy and apoptosis is complex and controversial. It varies with cell and stress distinction. Depending on the cellular context and stimulus, autophagy may be indispensable for apoptosis by turning on apoptosis. In other cellular settings, autophagy may rather antagonize or delay apoptosis, and inhibition of autophagy may increase the sensitivity of the cells to apoptotic signals. In some cell systems, two processes can occur independently [11,12,41]. In this study, apoptosis and autophagy were independent pathways in presences of Bax, which was triggered by TK VI. But apoptosis played a crucial role in the process. Plenty of signaling pathways overlaps are found between autophagy and apoptosis, including various kinases such as PI3K, PKB/Akt, Bcl-2 family members, PTEN, c-Myc, Ras and others [15,42]. Apoptosis was regulated by the Bcl-2 family (Bak, Bax and Bcl-xL) which conveyed the death message to mitochondria [26]. A report [10] demonstrated that *T. harzianum *peptaibols dissipated the mitochondrial membrane potential of human lung epithelial carcinoma cells, which accorded with our results (Figure 6C). In our study, ultrastructural characteristics might reveal that mitochondria lost membrane potential via a vacuolated manner, whereas, in typical apoptosis, the mitochondria maintained its shape and volume (Figure 5E). This was consistent with the report [24] that upon autophagic stimulation, depolarized mitochondria could promote sequestration and turnover of the damaged mitochondria in degradative vacuoles or autophagosomes. Our results showed that blockage of Bak would abrogate part of vacuolization effect induced by TK VI (Figure 5E, c). Cui Q [43] showed that physiological role Bak might be the suppression of autophagy. In contrast to previous studies, our results described a different activity of Bak during autophagy in TK VI-treated cells. The discovery heralded a new regulator involved in the induction of autophagy by calcium in response to TK VI. However, the mechanism of TK VI-induced vacuole formation still required further study. In HepG2 cells treated with TK VI, activated calpain induced subsequent translocation of Bax from cytosol to mitochondria. Then activated Bax predisposed to the release of cytochrome *c *and Smac, as well as clevage of caspase-3 and PARP. On the other hand, Buytaert and other researcher [44,45] demonstrated Bax proceeded to cell killing through apoptosis in its presence or via an autophagic pathway in its absence, which was similar to our results. Previous study [46] showed that moderate expression of Bcl-2 or Bcl-xL delayed apoptosis but did not prevent the rapid induction of autophagy. Our results exhibited changes in the ΔΨ_m _were retarded by overexpression of Bcl-xL, which resulted in a marked delay in the kinetics of PCD, whereas, the overexpression of Bcl-xL had no significant effect on autophagy.

**Figure 8:**
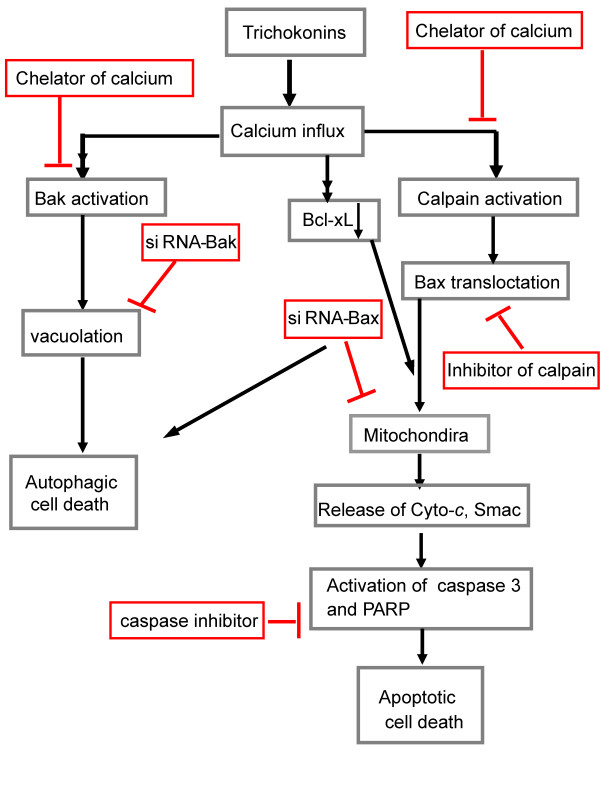
**Scheme of PCD pathway in HCC cells triggered by TK VI**. Ca^2+ ^entered through the cell membrane and activated Bak and calpain. The former ignited autophagy characteristic of cytoplasmic vacuolation and the latter promoted the translocation of Bax, which targeted mitochondria. On the other hand, Bcl-xL was downregulated almost in concurrence with the translocation of Bax. Bcl-xL overexpression retarded apoptosis, whereas it had no effect on authoagy. The release of Cyto-*c *and Smac from mitochondria into the cytosol was triggered by the preceding events, promoting the acivation of caspase-3 which subsequently led to PARP activation. Specific depletion of Bak expression could partly attenuate TK VI-induced autophagy. However, blockage of Bax led to increased autophagy.

Based on our results, a novel cell death pathway triggered by antibiotic peptaibol TK VI in HepG2 cells was proposed. TK VI-induced cell death might represent an intertwined type of autophagy and apoptosis, which was previously unknown. TK VI initially acted on the cell membrane, and then resulted in a Ca^2+ ^influx from extracellular medium. It both triggered the activation of Bak, which mainly involved in autophagy, as well as induced μ-calpain activation. Upon activation of Bax by calpain, Bax, in turn, translocated to the mitochondria, where it promoted loss of ΔΨ_m _and the release of cytochrome *c *and Smac/DIABLO. Finally, caspase-3, and PARP were activated, leading to apoptosis. Specific depletion of Bak expression could partly attenuate TK VI-induced autophagy. However, blockage of Bax led to increased autophagy (Figure 8). Antimicrobial peptides have been receiving increasing attention for their cytotoxic activity toward cancer cells that parallels their antimicrobial activity [47,48]. Our data suggested that peptaibols might be a new class of candidates for anti-tumor agents.

## Conclusions

In conclusion, this work demonstrated that TK VI induced growth inhibition of hepatocellular carcinoma cells (HCC cells) in a dose-dependent manner. However, at lower concentration, TK VI did not obviously impair the viability of normal liver cells. In our case, the targets of TK VI were related to Bax-apoptosis and Bak-autophagy pathway activation. Nevertheless, the validation of potential use of TK VI required further investigation on *in vivo *hepatocellular carcinoma models.

## Methods

### Reagents

Fluo-3 acetoxymethyl ester (Fluo-3 AM), acridine orange, 3-methyladenine (3-MA), anti-caspase-3, anti-PARP, anti-calpain, anti-Bax, anti-Smac, anti-Cyc-*c *and anti-Bcl-xl monoclonal antibodies were all purchased from Sigma (Sigma-Aldrich, St Louis, MO). The antibodies for detection of activated Bak were purchased from Millipore (Charlottesville, VA). DMEM and bovine calf serum were obtained from Gibco BRL (Grand Island, NY). benzyloxycarbonyl-Val-Ala-Asp-(OMe) fluoromethyl ketone (z-VAD-fmk) was obtained from Oncogene Research Products (San Diego, CA). The secondary antibody (goat anti-rabbit or rabbit anti-mouse Alexa 488) and 1, 2-Bis (O-aminophenoxy) ethane-N, N, N-, N-tetraacetic acid (BAPTA) acetoxymethyl ester were purchased from Molecular Probes, Inc. (Eugene, OR).

### Purification of Trichokonins *T. pseudokoningii*

SMF2 was fermented by solid-state fermentation and TK VI was purified using the methods as previously described [19,20]. The purified TK VI was dissolved in PBS.

### Cell culture, treatment and establishment of HepG2 cells stably expressing Bcl-xL

BGC-823, A549 and HepG2 cells were obtained from the Institute of Biochemistry and Cell Biology (Shanghai, China). They were grown in DMEM supplemented with 10% fetal calf serum under an atmosphere of 5% CO_2_. In this study, HepG2 cells were pretreated with the following compounds: the calpain inhibitor SJA6017 (100 μM), the caspase inhibitor z-VAD-fmk (200 μM), 3-MA (2 mM), and BAPTA (10 mM).

HepG2 cells were transfected with a pcDNA3.1 vector (Invitrogen, San Diego, CA) containing the coding sequence for Bcl-xL (pcDNA3.1/Bcl-xL), or with a control neomycin-resistant expression vector pcDNA3.1, using Lipofectin reagent (Invitrogen) according to the manufacturer's instructions.

### Assessment of apoptosis and autophagy

The MTT tetrazolium assay was performed as previously described to measure the density of viable cells [17].

Phosphatidylserine (PS) exposure was evaluated by using the Annexin V-FITC apoptosis detection kit (BioVision, Mountain View, CA) following the manufacturer's instructions. Annexin V binding was analyzed equipped with a FITC signal detector FL1 (Ex = 488 nm, Green) and PI staining with a phycoerythrin emission signal detector FL2 (Ex = 585 nm, Red) using the FACScan cytometer (Becton Dickinson, San Jose, CA). Percentage of apoptotic cells of the total (10^6 ^cells) was calculated using FlowJo 4.5.2 software.

DNA fragmentation assay was performed for the detection of DNA strand breaks. The detection was carried out according to the instructions for 3' end labeling of DNA *in situ *(Oncogene Research Products, San Diego, CA). Percentage of apoptotic cells of the total (10^6 ^cells) was calculated using FACScan cytometer. Additionally, cells were analyzed using NIKON ECLIPSE TE2000-E fluorescence microscope.

For the determination of DNA fragmentation in oligonucleosomal fragments (DNA laddering), total cellular DNA was extracted from 5 × 10^6 ^cells using the Easy-DNA kit (Invitrogen) following the instructions provided by the manufacturer. The obtained DNA was analyzed by electrophoresis on a 1.5% agarose gel.

MitoCapture™ (Biocarta, Hamburg, Germany) was used to evaluate changes in mitochondrial membrane potential (ΔΨ_m_). Cell suspensions (5 × 10^5^) were incubated at 37°C for 15 min in 1 ml PBS containing 1 mM rhodamine 123, and subsequently analyzed with a FACSCalibur flow cytometer.

The analysis of autophagy was undergone by MDC as described previously [21]. The percentage of autophagic cell death was analyzed using flow cytometry with acridine orange dyeing. The fluorescence emission of green (510~530 nm) and red (650 nm) from 1 × 10^6 ^cells was measured with a flow cytometer using CellQuest software. Moreover, the cells were exposed to 20 μM TK VI for 16 h and localization of LC3-II was detected as described by Herman [22].

### Cell morphological changes detected by electron microscopy

Ultrastructural change was detected using transmission electron microscopy with the method as described by Herman [22] and examined with a JEM-1230 transmission electron microscope (JEOL, Japan).

### Measurement of cytoplasmic calcium level

The changes in cytosolic calcium level ([Ca^2+^]_*c*_) were measured by using the cell-permeable Ca^2+^-sensitive fluorescent dye Fluo-3 acetoxymethyl ester (Fluo-3 AM). The cultures were treated for the designated time with the indicated concentrations of TK VI diluted in PBS alone or containing 2 mM CaCl_2_. After rinsing two times in HEPES-buffered saline, the live cells were placed in an open chamber (Molecular Probes, Inc., Eugene, OR) with 500 ml of HEPES solution and positioned on the stage of a Zeiss LSM 510 confocal laser scanning microscope equipped with an Argon laser. The raw fluorescence intensities (fluo-3) of user-selected areas were used for data analyses.

### RNA interference

For Bak, the sense and antisense strands of siRNA were, beginning at nucleotide 310, 5'-UGCCUACGAACUCUUCACCdTdT-3' (sense) and 5'-GGUGAAGAGUUCU AGGCAdTdT-3' (antisense). For Bax, the sense and antisense strands of siRNA were, beginning at nucleotide 217, 5'-UAUGGAGCUGCAGAGGAUGdTdT-3' (sense) and 5'-CAUCCUCUGCAGC UCCAUAdTdT-3' (antisense). Non-specific control siRNA (target 5'-GCATTGTATGCGATCGCAGAC-3') was served as a control. The transfection of siRNA oligonucleotides was performed with Lipofectamine 2000 (Invitrogen) according to the manufacturer's recommendations. After 48 h, the cells were treated with TK VI for the indicated times. At the end of treatment, cells were harvested for experiments.

### Western blot

Proteins in mitochondrial and cytosolic fractions were extracted using cytochrome *c *releasing apoptosis detection kit (Biovision, Mountain View, CA). Protein concentration was determined using a BCA protein assay kit (Bioteke). Equal amount of proteins (20 μg) was loaded onto SDS-PAGE gels and the proteins were electrically transferred to a PVDF film (Amersham, Ayesbury, UK). Immunoblots were analyzed using specific primary antibodies against Bcl-xl, calpain, Bax, Bak, LC3, caspase-3, PARP, Smac, Cyc-*c *and β-actin, and proteins were visualized using an advanced chemiluminescence detection kit (Amersham, Ayesbury, UK). β-actin was used for internal control to confirm that the amount of protein was equal.

### Immunohistochemical studies

HepG2 cells (5 × 10^4^) were seeded in 12-well plates. After being washed with PBS, cells were incubated with 100 nM MitoTracker for 30 min and fixed with ice-cold 4% paraformaldehyde. The fixed cells were subsequently incubated with the primary antibody rabbit anti-Bak and Smac (1:500) or mouse anti-Bax and cytochrome *c *(1:200) in blocking solution (1.5% bovine serum albumin in TBST) for 1 h at room temperature, and then stained with FITC-conjugated anti-mouse and anti-rabbit IgG, respectively. Cells were examined under a Zeiss LSM 510 confocal laser scanning microscope. Data was representative of at least three independent experiments.

### Statistical analysis

All results were expressed as mean ± SD. P < 0.05 was considered statistically significant. All statistical analyses were performed using SPSS 11.5 for Windows.

## Competing interests

The authors declare that they have no competing interests.

## Authors' contributions

MS performed the major part of experiments, statistical analysis and drafted the manuscript. HNW and STX participated to cell cultures, cell proliferation assays and assessment of apoptosis and autophagy. YL conducted measurement of cytoplasmic calcium level and determination of DNA fragmentation in oligonucleosomal fragments. CYS provided fungal strain and purification of TK VI. XLC contributed to the final drafting and critical revision of the manuscript. YZZ conceived of the study, participated in the design of the experiments and helped to revise the manuscript. All authors read and approved the final manuscript.

## Supplementary Material

Additional file 1 **Identification of strain SMF2**. Strain SMF2 grew on PDA (potato dextrose agar) medium plate at 28°C in darkness and its morphological character was observed. The 18S rDNA and ITS (internal transcribed spacer) sequences of strain SMF2 were amplified from its genomic DNA by PCR. The sequence of 18S rDNA and ITS gene were, respectively, blasted in GenBank database. Strain SMF2 was named as *T. pseudokoningii *SMF2 based on its colony and conidiophore morphology as well as its 18S rDNA and ITS sequences. Click here for file
