# Supplementary material for: Antimicrobial peptaibols, novel suppressors of tumor cells, targeted calcium-mediated apoptosis and autophagy in human hepatocellular carcinoma cells
Source: Mol Cancer. 2010 Feb 2;9:26. doi: 10.1186/1476-4598-9-26 (PMC2825246; doi:10.1186/1476-4598-9-26)
Supplement: Additional file 1 — Identification of strain SMF2. Strain SMF2 grew on PDA (potato dextrose agar) medium plate at 28°C in darkness and its morphological character was observed. The 18S rDNA and ITS (internal transcribed spacer) sequences of strain SMF2 were amplified from its genomic DNA by PCR. The sequence of 18S rDNA and ITS gene were, respectively, blasted in GenBank database. Strain SMF2 was named as T. pseudokoningii SMF2 based on its colony and conidiophore morphology as well as its 18S rDNA and ITS sequences. [file 1476-4598-9-26-S1.DOC]

**Identification of strain SMF2**

Strain SMF2 grows fast on PDA (potato dextrose agar) medium plate at 28^o^C in darkness. The colony is white in the first 2-day cultivation. After 3 days, colony radius on PDA medium is among 55-65 mm, and it tends to form in aerial mycelium in a single ring. After 96 h, a pale yellow pigment diffuses through the agar, and no odor is noticed (Fig. S1). SEM observation showed that strain SMF2 loosely aggregates into fascicles (Fig. S1a). The conidia are green, oblong, smooth and dry, with the size of 3.5-6.0 (Length) × 2.2-3.0 (Width) µm (L/W =>1.3) (Fig. S1b). It produces conidiophore, which are mainly secondarily branched. Main branches of the conidiophores produce lateral side branches, some of which rebranch and terminate in phialides. The primary and secondary branches arise at about 90^o^C with respect to the main axis (Fig. S1c). The mycelium is white and septate (Fig. S1d). In general, the morphology of strain SMF2 is similar to that of *Trichoderma* *pseudokoningii* (<http://nt.ars-grin.gov/taxadescriptions/keys/TrichodermaIndex.cfm>).

The 18S rDNA and ITS (internal transcribed spacer) sequences of strain SMF2 were amplified from its genomic DNA by PCR with primers shown in Table S1, and were submitted to GenBank with the Accession No. **FJ605099**. The sequence of 18S rDNA and ITS gene were, respectively, blasted in GenBank database, and the blast results were shown in Table S2 and S3. Both the 18S rDNA and ITS sequences showed 100% identity with that of *T. pseudokoningii.* Therefore, based on its colony and conidiophore morphology as well as its 18S rDNA and ITS sequences, strain SMF2 was named as *T. pseudokoningii* SMF2.


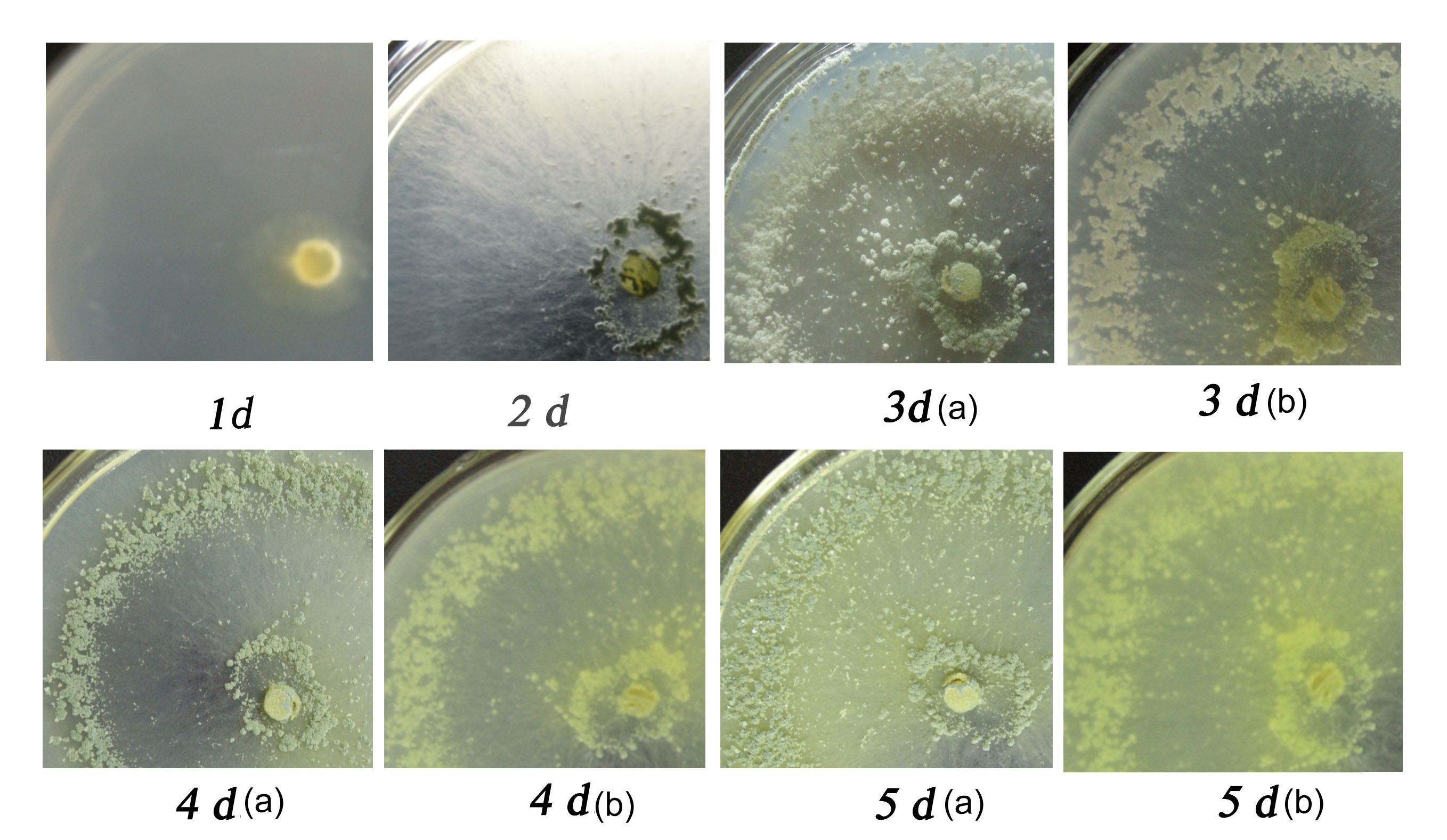


Fig. S1. Colony of strain SMF2 on PDA plate after cultivation at 28^o^C for 1, 2, 3, 4 and 5 d. (a) Frontal view of the colony; (b) Reversed view of the colony.


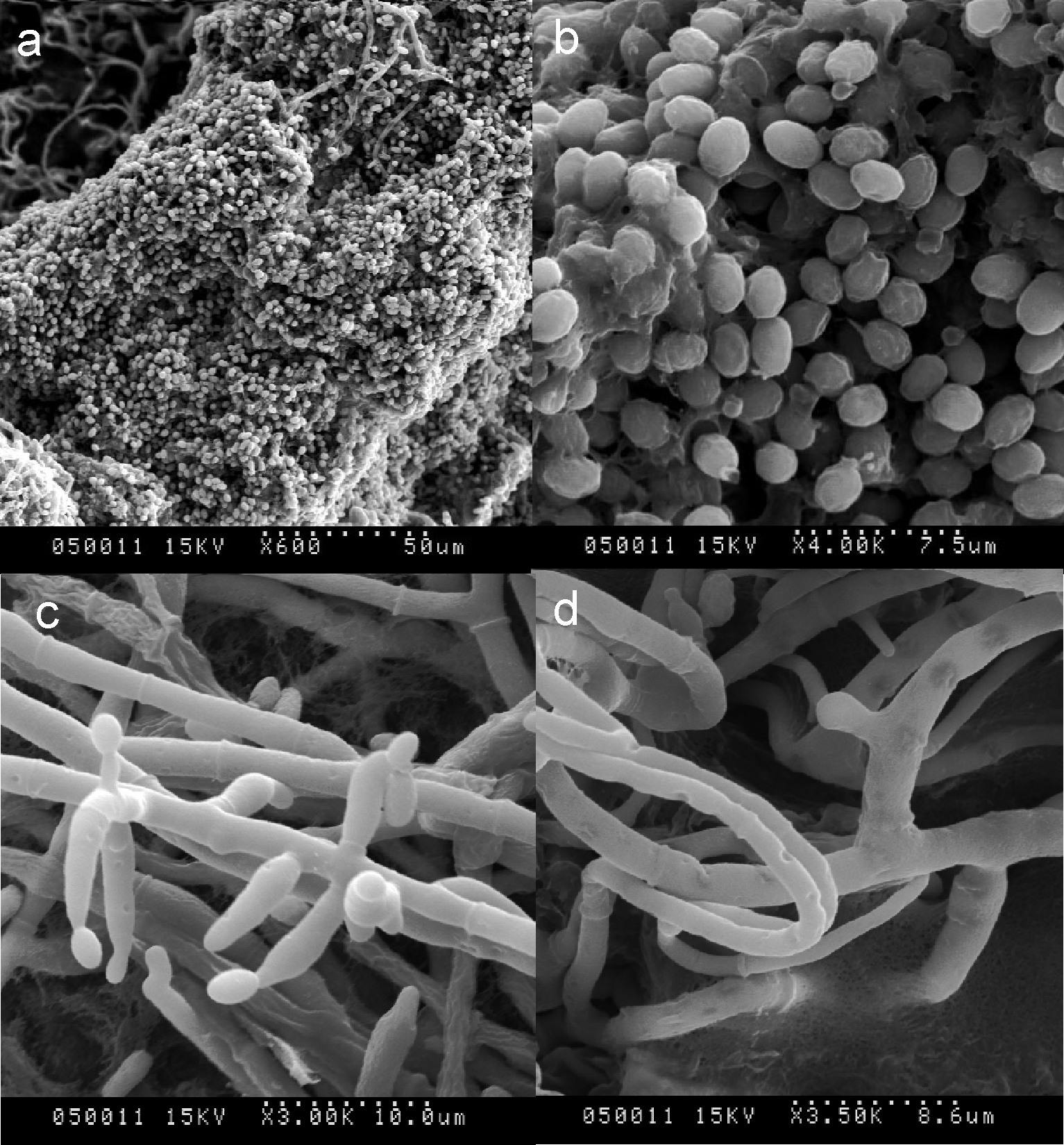


Fig. S2. Conidia aggregation (a), Conidia (b), Conidiophore (c), and mycelium (d) morphology of *Trichoderma* spp. SMF2 observed by scanning electron microscope. Strain SMF2 was cultured on PDA medium at 28°C for 3 d.

Table S1. Primer sequences for cloning of 18SrDNA and ITSs gene of strainSMF2*

| Primers | Sequences (5’-3’) |
| --- | --- |
| NS1 | GTAGTCATATGCTTGTCTC |
| NS2 | GGCTGCTGGCACCAGACTTGC |
| NS3 | GCAAGTCTGGTGCCAGCAGCC |
| NS4 | CTTCCGTCAATTCCTTTAAG |
| NS5 | AACTTAAAGGAATTGACGGAAG |
| NS6 | GCATCAGACCTGTTATTGCCTC |
| NS7 | GAGCAATAACAGGTCTGTGATGC |
| NS8 | TCCGCAGGTTCACCTACGGA |
| ITS1 | TCCGTAGGTGAACCTGCGC |
| ITS2 | GCTGCGTTCTTCATCGATGC |
| ITS3 | GCATCGATGAAGAACGCAGC |
| ITS4 | TCCTCCGCTTATTGATATGC |

*Primers NS1-NS8 were used for cloning of the 18SrDNA gene of strain SMF2, and primers ITS1-ITS4 were used for cloning of the ITS gene of strain SMF2.

Table S2. Partial results of the 18S rDNA sequence of strain SMF2 aligned with that of a number of *Trichoderma* strains in the GenBank/EMBL.

| Accession | Description | Total score/E value | Max Identities |
| --- | --- | --- | --- |
| AF548104 | *T. viride strain* ALI 210 | 3096/0.0 | 100% |
| AF548104 | *T. pseudokoningii* S-38 | 3096/0.0 | 100% |
| AF510497 | *Hypocrea jecorina* | 3085/0.0 | 99% |
| AF548103 | *H. jecorina* RutC-30 | 3072/0.0 | 99% |
| AF548102 | *H. jecorina* QM9414 | 3072/0.0 | 99% |
| EU722404 | *H. koningii* JH | 3057/0.0 | 100% |
| AY489694 | *H. rufa* GJS89-127 | 3051/0.0 | 100% |
| AF548100 | *T. harzianum* ALI232 | 3038/0.0 | 99% |

Table S3. Partial results of the ITS sequence of strain SMF2 aligned with that of a number of *Trichoderma* strains in the GenBank/EMBL.

| Accession | Description | Total score/E value | Max Identities |
| --- | --- | --- | --- |
| FJ462769 | *T. longibrachiatum* isolate T61 | 1011/0.0 | 100% |
| DQ026019 | *T. pseudokoningii* AHS-515-28 | 1011/0.0 | 100% |
| DQ000625 | *H. jecorina* strain ATCC 24449 | 1011/0.0 | 100% |
| X93966 | *H. schweinitzii* | 1011/0.0 | 100% |
| EF568085 | *T. viride* strain WM06.336 | 1005/0.0 | 100% |
| EF552703 | *H. lixii* | 1005/0.0 | 100% |
| AF359258 | *T. harzianum* | 1005/0.0 | 100% |
| AJ606397 | *H. orientalis* | 1003/0.0 | 100% |
| EU520125 | *T. viride* | 1002/0.0 | 99% |
| EU520243 | *H. koningii* | 1000/0.0 | 98% |
